# Donations: how to ensure you really benefit

**Published:** 2010-09

**Authors:** Ismael Cordero, Neil Murray, Henry E Nkumbe

**Affiliations:** Senior Clinical Engineer, ORBIS International, 520 8th Ave, 11th Floor, New York, NY 10018, USA.; Medical Advisor, CBM West Africa. Email: n.murray.cbm@gmail.com; Medical Advisor, CBM Madagascar, SALFA Eye Project Antananarivo, PO Box 3825, Antananarivo 105, Madagascar.

**Figure:**
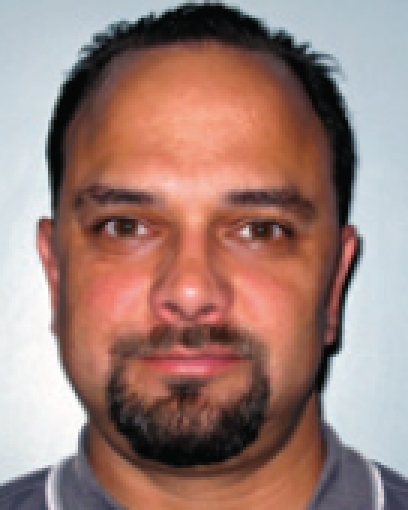


**Figure:**
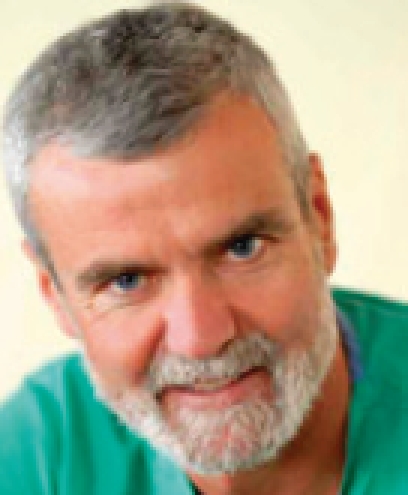


**Figure:**
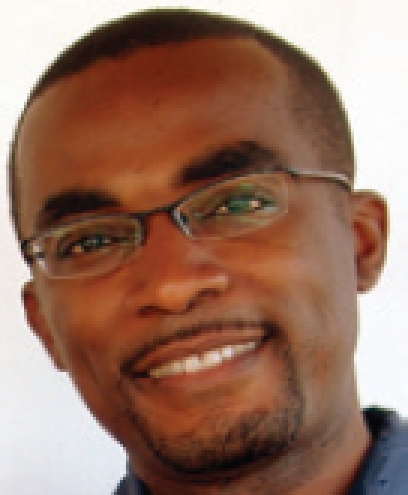


Appropriate donated equipment or consumables can help to achieve the goals of VISION 2020. However, according to the World Health Organization (WHO) Guidelines on Healthcare Equipment Donation (2000), an estimated 70% or more of donated medical equipment is not being used.

## Why do things sometimes go wrong?

Some of the causes are:

- Inadequate communication between the donor of the equipment and the receiving eye unit, so that the eye unit ends up with an item which is not appropriate, is not robust, cannot be maintained, does not include spare parts or a manual, and which no-one knows how to use.
- Failure to identify all the costs involved, such as training, shipping and clearing, and operation and maintenance.

## Before you accept a donation

Think about the following:

- Is this something you really need, and is it the best use of scarce resources? For example, has the donation of sophisticated devices (phaco machines, lasers, or ultrasound machines) been balanced against the need for basic public health equipment such as ophthalmoscopes?
- Is it the right tool for the job? Is it an appropriate make and model? Refer to the *IAPB Standard List for a VISION 2020 Eye Care Service Unit 2010* (Standard List). See page 36.
- Does your organisation have the necessary resources and skills to make good use of it?
- Do you have the budget to use and maintain the equipment in the long term? You will have to budget 3-6% of the purchase price of the donated equipment per year for consumables, parts, maintenance, and user training. This may or may not form part of the donation.
- Is the potential donor a credible individual or institution? Talk to others who have received donations from them. Give preference to items from companies with an established track record for after-sales service in the country or sub-region; most of these companies are in the Standard List.

## Talking to a potential donor

Explain your needs, and how the requested equipment or consumables will meet these needs. Describe:

- Why the resources presently available are not satisfactory
- What specific interventions the requested equipment or consumables will be used for
- Which and how many procedures will be performed using them
- How they will help you meet the expected demand.

If your donor is overseas or is likely to import the equipment or consumables, first ensure these cannot be purchased locally at competitive prices. Encouraging donors to buy locally will help develop local markets and will make it easier to get direct support from the vendors without needing to involve the donor.

Show that you are a credible organisation. You could do this by providing a list of references (your ministry of health, non-governmental organisations, charities, or religious institutions) which the donor can contact.

**TIP:** Ask for photographs of the equipment you are requesting, where possible. This will reduce confusion and possible waste, as equipment and instruments may have different names depending on where a person was trained, particularly in non-English speaking countries. Many projects have requested instruments and equipment that were never used because they asked for the wrong item.

**‘Explain your needs, and how the requested equipment or consumables will meet these needs’**

## Questions to ask the donor

As mentioned in our article on purchasing (page 34) getting a new item of equipment is not a simple matter. Equipment may require some or all of the following:

- Installation and user training
- Regular preventative maintenance
- Breakdown support
- Accessories and spare parts
- Supporting materials
- Electricity and water supply.

The questions below should help you cover all the most important aspects of the donation with your prospective donor.

### 1. Installation

Who will be able to install the equipment? If your eye unit does not have the skills and resources required, is the donor willing to arrange and pay for installation? Who will train the people who will use the equipment?

### 2. Regular preventative maintenance

How will the equipment be maintained on a regular basis? Will this mean training the people who will be responsible? If so, how can this be achieved? Ideally, training in maintenance should be given at the time of installation, although it is also possible to arrange training with a similar piece of equipment in a neighbouring eye unit before the donated equipment arrives.

Especially with very expensive, unfamiliar, or sophisticated equipment, some users may feel reassured if an experienced user were available to demonstrate proper assembly, use, and routine maintenance. Short of this, complete documentation in the local language (including circuit diagrams for local maintenance technicians and engineers) should be made available.

### 3. Breakdown support

Despite your best efforts to keep equipment in good working order, breakdowns may occur from time to time. Is there an agent in the country and within easy reach to undertake the repair or perform preventive maintenance and calibration services? If yours is to be the only piece of equipment of this make and model in the country, or if breakdown support is non-existent or very expensive, you should discuss these issues with the donor. Would it be best to refuse the donation and ask for an item of a different make or model?

**Figure:**
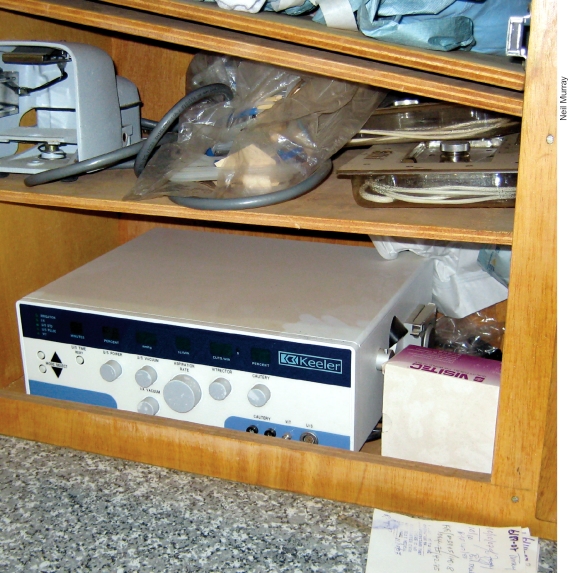
This donated phaco machine has ended up in the back cupboard of an operating theatre. It was never used because of a lack of parts and problems with the power supply. SIERRA LEONE

Neil Murray

Especially with sophisticated and expensive equipment, preference should be given to companies with established track records for after sales service in the country or sub-region.

### 4. Accessories and spare parts

What essential accessories and consumables will be required, such as cables, reagents, filters, electrodes, and recording paper? Are they being donated? If not, can they be obtained locally and does your eye unit have the budget to buy them?

Does the donation include the necessary spare parts? If not, are spare parts easily available locally, and can you afford the monthly or yearly costs?

You can request that donors include commonly required spares (such as replacement bulbs, fuses, etc.) in the donation, as well as any other items that wouId take longer than six months to be delivered.

### 5. Supporting materials

Above all, you need to check that the donated item comes complete with all appropriate installation instructions and operating manuals in a language you can understand. Ideally, there should be expertise locally to support the installation and maintenance, but it is worth asking the donor whether there is also a remote troubleshooting facility such as an internet or telephonic technical help desk.

### 6. Electricity and water supply

Has the equipment been fitted with all the devices it needs to work in your location? For example, electrical equipment needs the correct electrical plugs, voltage surge protectors, and other devices necessary to ensure regular, uninterrupted power supply (see ‘top tips’ on page 27).

If the item of equipment requires running water, will this be available? If not, is the equipment really suited to your circumstances?

## Shipping and clearing donated equipment

You need to be sure that the donated equipment or consumables are shipped with a recent biomedical certification and a minimum one-year guarantee.

The donation should be packed and shipped in accordance with international shipping regulations, with appropriate packing for the donation and mode of transport. Documents must list everything in the shipment and clearly indicate that it is a donation. You will need to check the customs regulations in your country and inform the donor in advance, before shipping, to reduce the risk of high clearance charges and delays, which may result in extra charges by the shipping company (demurrage).

## After receiving the donation

Make sure you read and understand the user manual and then familiarise yourself with the equipment before attempting to assemble or operate it.

Ensure that each item of equipment has a maintenance plan that is respected and followed, and that there is a budget allocated for maintenance each year.

Keep the donors informed about both the successes and challenges with the donated equipment - this will help you to build a positive, and hopefully long-term, relationship.

In conclusion, maintaining open communication with your donor and following these guidelines should ensure that your donation goes smoothly. With time, your eye unit will be able to demonstrate that the donations have resulted in improved services that bring satisfaction to patients, staff, and the donor.

**If you are a donor**

**Develop a relationship with the eye unit.** What do they need? Find out what equipment is best suited to their work and environment. This depends not just on the durability and functionality of the equipment, but also on the eye unit itself- does the unit have staff with the knowledge and skills needed to use the equipment? Can the unit afford the operation and maintenance of the equipment?

**Balance need and quality.** Although it is not appropriate to donate out-of-date equipment, an eye unit in a low- or middle-income country may be able to use equipment that is no longer considered suitable in a high-income setting. However, it is your responsibility to ensure that the equipment meets the eye unit's needs and that maintenance and repair support, as well as spare parts, will be available for each donated item for the next 5-10 years.

